# Analysis of differential gene expression of pro-inflammatory cytokines in the nasopharyngeal milieu of mild & severe COVID-19 cases

**DOI:** 10.1371/journal.pone.0279270

**Published:** 2022-12-30

**Authors:** Unzela Ghulam, Fizza Nazim, Nida Farooqui, Syed Rizwan-ul-Hasan, Muhammad Faraz Anwar, Khalid Ahmed, Abid Jamal, Hammad Afzal Kayani, Nouman Mughal, Azhar Hussain, Antonio Sarria-Santamera, Syed Hani Abidi

**Affiliations:** 1 Department of Biosciences, Shaheed Zulfikar Ali Bhutto Institute of Science and Technology, Karachi, Pakistan; 2 Department of Biological and Biomedical Sciences, Aga Khan University, Karachi, Pakistan; 3 Department of Computer Science, DHA Suffa University, Karachi, Pakistan; 4 Department of Biochemistry, Bahria University Medical & Dental College, Karachi, Pakistan; 5 Cancer Foundation Hospital, Karachi, Pakistan; 6 Department of Surgery, Aga Khan University, Karachi, Pakistan; 7 Department of Biomedical Sciences, Nazarbayev University School of Medicine, Astana, Kazakhstan; Texas Christian University, UNITED STATES

## Abstract

**Introduction:**

A subset of individuals with COVID-19 can suffer from a severe form of the disease requiring breathing support for respiratory failure and even death due to disease complications. COVID-19 disease severity can be attributed to numerous factors, where several studies have associated changes in the expression of serum pro-inflammatory cytokines with disease severity. However, very few studies have associated the changes in expression of pro-inflammatory changes in the nasopharyngeal milieu with disease severity. Therefore, in the current study, we performed differential gene expression analysis of various pro-inflammatory cytokines in the nasopharyngeal milieu of mild & severe COVID-19 cases.

**Material and method:**

For this retrospective, cross-sectional study, a total of 118 nasopharyngeal swab samples, previously collected from mild and severe (based on the WHO criteria) COVID-19 patients were used. A real-time qPCR was performed to determine the viral loads and also evaluate the mRNA expression of eight cytokines (*IL-1*, *IL-2*, *IL-4*, *IL-6*, *IL-10*, *IFN-γ*, *TGF-β1*, *and TNF-α*). Subsequently, an unpaired T-test was applied to compare the statistical difference in mean expression of viral loads and each cytokine between the mild and severe groups, while the Pearson correlation test was applied to establish a correlation between disease severity, viral load, and cytokines expression. Similarly, a multivariable logistic regression analysis was performed to assess the relationship between different variables from the data and disease severity.

**Results:**

Out of 118 samples, 71 were mild, while 47 were severe. The mean viral load between the mild and severe groups was comparable (mild group: 27.07± 5.22; severe group: 26.37 ±7.89). The mRNA expression of cytokines *IL-2*, *IL-6*, *IFN- γ*, and *TNF-α* was significantly different in the two groups (p<0.05), where the Log_2_ normalized expression of *IL-2*, *IL-6*, *IFN- γ*, and *TNF-α* was found to be 2.2–, 16–, 2.3–, and 1.73–fold less in the severe group as compared to the mild group. Furthermore, we also observed a significant positive correlation between all the cytokines in the severe group. The multivariate analysis showed a significant relationship between age, *IL-6*, and disease severity.

**Conclusion:**

This decreased expression of certain cytokines (*IL-2*, *IL-6*, *TNF-α*, and *IFN-γ*) in the nasopharyngeal milieu may be considered early biomarkers for disease severity in COVID-19 patients.

## Introduction

The novel coronavirus (SARS-CoV-2) infection is one of the most serious public health issues at the current time. This virus may result in severe pneumonia with the onset of multiple other illnesses [1]. Clinical features of SARS-CoV-2 infection are similar to that of SARS-CoV, which include fever, dry cough, lethargy, etc. [2].

SARS-CoV-2 enters the host cell through the ACE2 receptor and the proteolytic activity of Transmembrane protease, serine 2 (TMPRSS2). The innate immune response is the initial defense against SARS-CoV-2 infection, which stimulates certain interferons, pro-inflammatory cytokines, and chemokines, as well as coordinates the activation of the adaptive immune response against the virus [3]. The host’s response is influenced by a variety of characteristics, including health status, age, and gender [4]. To develop an antiviral response, innate immune cells recognize the pathogen-associated molecular patterns (PAMPs) found in the form of viral genomic RNA, or act as intermediate during viral replication such as double-stranded RNA (dsRNA), which are identified either by endosomal RNA receptors (Toll-like receptor 3 (TLR3) and Toll-like receptor 7 (TLR7)) or by the cytosolic RNA sensor Retinoic acid-inducible gene I (RIG-I) and melanoma differentiation-associated gene 5 (MDA5) [5]. This event also activates the down streaming cascade signaling through the Interferon regulatory factor 3 (IRF-3) and Nuclear factor kappa-light-chain-enhancer of activated B cells (NF-κB) pathway, resulting in the translocation of IRF-s/NF-kB to the nucleus [6]. These transcription factors upregulate the expression of type I interferon and other pro-inflammatory cytokines. Additionally, through the interferon-α/β receptor (IFNAR), type 1 interferon (IFN) stimulates the JAK-STAT pathway, where STAT 1 and 2 are phosphorylated by JAK1 and Non-receptor tyrosine-protein kinase (TYK2) [7]. STAT1 and 2 bind with Interferon regulatory factor 9 (IRF9) and synergistically move into the nucleus to start the transcription of IFN genes under the control of a promoter containing IFN–stimulating response elements (ISREs) [8]. A cytokine storm, inflammation, and vascular disruption might result from excessive activation of the innate immune response, resulting in the wide range of symptoms seen in COVID-19 individuals [9].

Recent studies have shown that overexpression of pro-inflammatory cytokines (e.g. IL-6, IL-12, IFN- γ, IL1B, monocyte chemoattractant protein-1 (MCP-1), and interferon gamma-induced protein 10 (IP-10) in serum is strongly correlated with multiple organ failure in patients with COVID-19 disease [10]. Similarly, another study showed high levels of IP10, MCP1, granulocyte colony-stimulating factor (GCSF), Macrophage inflammatory protein-1 alpha (MIP-1α), and TNF-α in patients who required ICU admittance, indicating that the severity of the infection was correlated with the cytokines [11].

While most of these studies have focused on cytokine changes in serum, few have associated early cytokine changes following viral infection of the nasopharyngeal region [12], with disease severity. One study from China showed that pro-inflammatory cytokines/chemokine can be detected in the nasopharyngeal swab sample, where levels of cytokines can be valuable for the diagnosis and prognosis of systemic disease [13]. This suggests that the expression of certain cytokines in the nasopharyngeal samples may serve as early biomarkers predicting the severity of the disease. In this study, therefore, we performed differential gene expression analysis of various pro-inflammatory cytokines in the nasopharyngeal milieu of mild & severe COVID-19 cases.

## Methodology

### Sample collection and characterization of the sample as mild and severe based on the patient’s symptoms

For this retrospective, cross-sectional study, a total of 118 nasopharyngeal swab samples, previously collected from mild and severe (based on the WHO criteria [14]) COVID-19 patients were used after obtaining written informed consent from the patients. For nasopharyngeal sample collection, a flocked swab was inserted into the patient’s nostril until it reaches the nasopharynx, and the swab was then rotated a few times and left in the nasopharynx for approximately 5–10 seconds. The swab was taken out and immediately placed in viral transport media and stored at -80°C until further use. The samples were procured, respectively, from the Aga Khan University Hospital SARS-CoV-2/COVID-19 sample biorepository, and Cancer Foundation Hospital, Karachi-Pakistan. The patients with fever, cough, sore throat, etc., with no requirement of oxygen therapy or oxygen by mask or nasal cannula, were categorized in the mild group; whereas samples from patients requiring non-invasive ventilation (<93% oxygen saturation at rest) or invasive mechanical ventilator without other organ support, or invasive mechanical ventilation with other organ support were categorized in the severe group [15]. In the mild group, none of the patients were hospitalized and swabs/samples were collected as part of the COVID diagnostic test upon the onset of symptoms; while in the severe group, most of the swabs/samples were collected at the time of patient’s visit to Aga Khan University Hospital’s Emergency Department upon worsening of symptoms. However, there were eight cases where the PCR test was found to be positive, a few days prior to the patient’s hospital admittance. This study was approved by the Ethics Review Committee, Aga Khan University Hospital (ERC# 2021-5456-15382). Written consent forms were taken from each patient. To ensure the patient’s confidentiality the sample were given unique IDs.

### RNA extraction and cDNA synthesis

From all the SARS-CoV-2 positive nasopharyngeal samples, viral RNA was extracted using QIAamp viral RNA kit (Qiagen, Hilden, Germany) following the manufacturer’s instructions. The extracted RNA was stored at -80C till further use. From each sample, 2.5 μg RNA/20 μl of cDNA synthesis reaction was reverse transcribed using ONE SCRIPT PLUS cDNA Synthesis Kit (CAT # G236) from Applied Biological Materials inc. (ABM) following the manufacturer’s instructions and as described previously [16].

### qPCR for estimation of viral loads and cytokines gene expression

The viral load was determined using the COVID-19 genesig® Real-Time PCR assay (Primer design). Each 20μL triplex reaction mix containing 8μL of eluted RNA, and 12μL of the master mix was subjected to the following thermocycling conditions: 55°C for 10 min, 95°C for 2 min followed by 45 cycles of 95°C for 10 s, 60°C for the 60s. Ct values of Internal control and Target (ORF1ab) genes were measured on Hex and FAM channels, respectively, on Rotor Gene™ 3000 (Qiagen, USA). Viral load was plotted as Ct values.

The cDNA samples were used in the qPCR assay to estimate the expression level of each of the cytokine’s genes using gene-specific primers (S1 Table). The β-actin gene was used as a housekeeping gene to normalize the gene expression. For qPCR analysis, 2μl of cDNA was mixed with 4ul of BlasTaq 2X qPCR Mastermix (Cat # G891; ABM), and 0.5μl of each reverse and forward primers were added. The qPCR was performed using the following thermal cycling conditions: 95°C for 3 minutes, 40 cycles of 95°C for 15 seconds, and 57.8°C to 64°C (depending on the primer) for 1 minute with a melt curve at 55-95C. All reactions were run in duplicate. The delta Ct method was used to plot/compare the expression of each cytokine in both groups, while 2^(-ΔΔCt)^ methods were used to estimate the relative expression/fold change of each cytokine in the severe vs mild group [17, 18].

An unpaired T-test was applied to compare the statistical difference in mean expression of viral loads and each cytokine between the mild and severe groups. We also applied the Pearson correlation test to establish a correlation between cytokine expression. Furthermore, a multivariable logistic regression analysis was performed with all the variables from the data set using ‘severity’ as the outcome variable. In all tests, a p<0.05 was considered significant. IBM SPSS software, version 20 was used for statistical analyses.

## Results

### Viral loads in mild and severe groups and correlation between viral loads and disease severity

**Fig 1:**
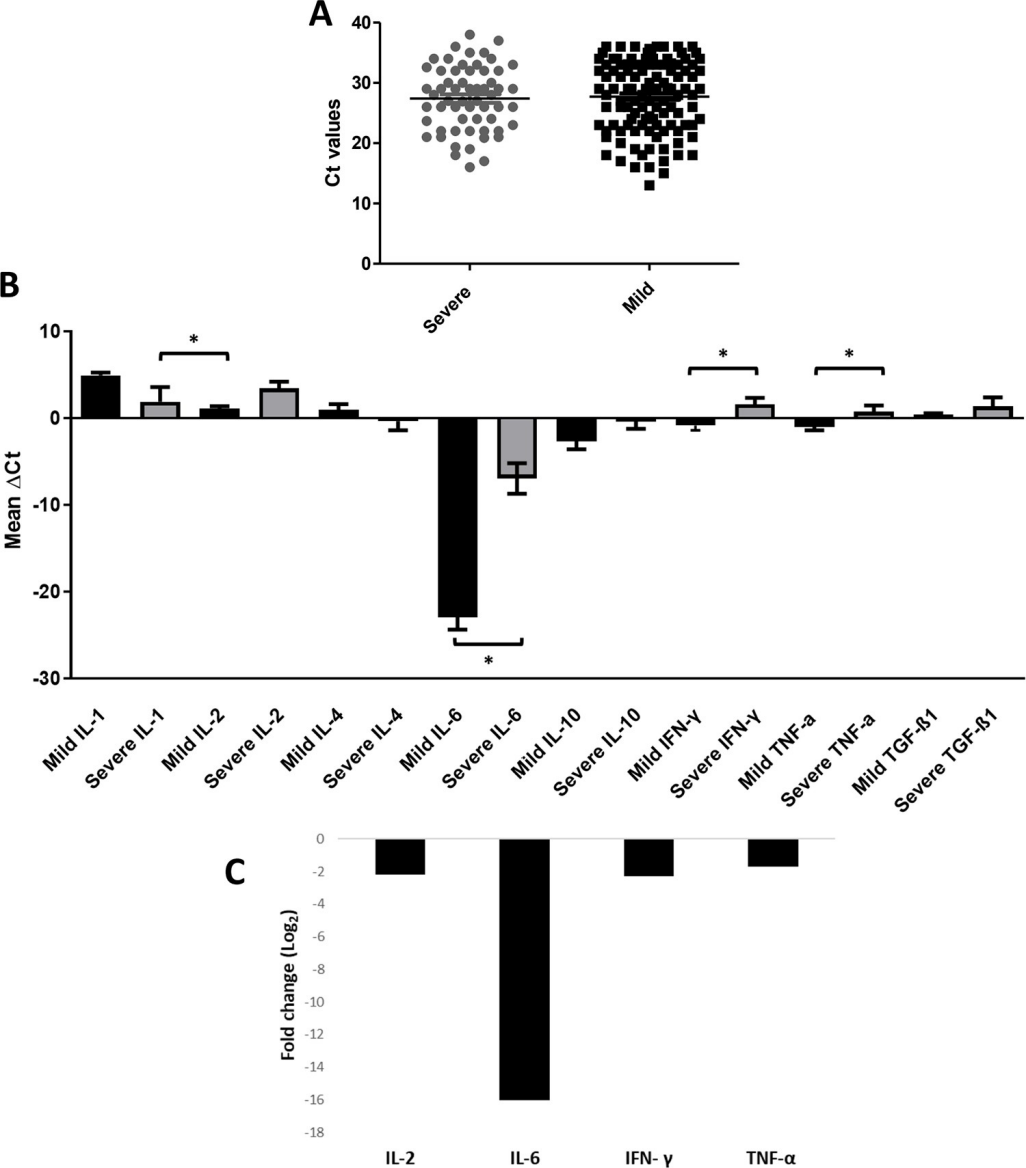
Viral loads and cytokine expression in mild vs severe group. The figure shows **A)** viral loads (Ct values) distribution, **B)** expression of cytokines (mean ΔCt values) in mild and severe groups, while **C)** shows Log2 normalized fold change (2^(-ΔΔCt)^) in cytokine expression in severe groups relative to the mild group. **B)** The line above bars with an asterisk sign indicates a significant difference (p<0.05) in the expression of the tested genes between the mild and severe groups.

For this study, a total of 118 nasopharyngeal swabs samples in viral transfer medium were collected, out of which, 71 samples were from patients with mild symptoms (ranging from mild flu to sore throat), while 47 samples were from patients with severe symptoms (ranging from fever to critical covid pneumonia) (Table 1). The mean viral loads for the mild and severe groups were (27.07± 5.22) 10 copies/ml and (26.37 ±7.89) 10 copies/ml, respectively (Fig 1A). Statistically, no significant difference was found between the two groups, and no statistically significant correlation was found between viral loads and disease severity.

**Table 1:** Demographic data. The demographic data of 118 participants from the SARS COV2 patients.

Variables	Category	Percentage total (n = 118)
**Age**	1–19	7 (6.0%)
	20–39	21 (17.7%)
	40–70	90 (76.2%)
**Sex**	Male	76 (64.4%)
	Female	42 (35.5%)
**Disease severity**	Mild	71 (60%)
	Severity	47 (39.8%)

### Differential expression of cytokines between the mild and severe group and correlation between cytokine expression and disease severity

Analysis of differential expression of cytokines in the mild and severe groups showed the expression of *IL-2* (severe: 3.41±0.77, mild: 1.11±0.23 Mean ± S.E), *IL-6* (severe: -6.95±1.71, mild: -22.95±1.41 Mean ± S.E), *TNF-α* (severe: 0.72±0.7, mild: -1.01±0.40 Mean ± S.E), and *IFN-γ* (severe: 1.57±0.75, mild: -0.85±0.57 Mean ± S.E) to be significantly (p<0.05) different in the two groups (Fig 1B), where the Log2 normalized expression of *IL-2*, *IL-6*, *IFN- γ*, and *TNF-α* was found to be 2.2–, 16–, 2.3–, and 1.73–fold less in the severe group as compared to the mild group. (p<0.05; Fig 1C).

In the next step, the Pearson correlation test was applied to investigate the relationship between cytokine expression in mild and severe groups (Table 2). In the mild group, a statistically significant weak positive correlation was found between *IL-2* and *IL-4* (r = 0.403, p = 0.00), *IL-2* and *TGF-β1* (r = 0.334, P = 0.004), *IL-1* and *IL-6* (r = 0.31, p = 0.01), and *IL-10* and *IL-4* (r = 0.403, p = 0.00). In the severe group, a significant moderate to strong positive correlation was observed between all the cytokines, except *IL-6* (Table 2).

**Table 2:** Correlation between cytokine expressions in the mild and severe groups. Each column shows the coefficient of correlation (r). Correlations with significant p-values (p<0.05) are shown in bold.

**Mild group**								
**Cytokines**	***IL- 1***	***IL-2***	***IL-4***	***IL-6***	***IL-10***	***IFN- γ***	***TNF—α***	***TGF-β1***
*IL- 1*	-	0.01	0.11	**0.31**	0.19	0.01	0.19	-0.02
*IL-2*	0.01	-	0.40	-0.11	0.22	0.13	0.22	0.33
*IL- 4*	0.11	**0.40**	-	-0.14	**0.46**	0.78	-0.01	-0.17
*IL-6*	**0.31**	-0.11	-0.14	-	0.14	-0.14	0.12	0.18
*IL-10*	0.19	0.22	0.46	0.14	-	0.03	0.31	-0.14
*IFN-γ*	0.01	0.13	0.78	-0.14	0.03	-	-0.03	0.09
*TNF- α*	0.19	0.22	0.12	-0.14	0.03	-0.03	-	0.12
*TGF-β1*	-0.02	**0.33**	- 0.17	0.18	-0.14	0.09	0.12	-
**Severe group**								
**Cytokines**	***IL- 1***	***IL-2***	***IL-4***	***IL-6***	***IL-10***	***IFN- γ***	***TNF-α***	***TGF-β1***
*IL- 1*	-	**0.45**	**0.37**	-0.10	**0.45**	**0.54**	**0.50**	**0.37**
*IL-2*	**0.45**	-	**0.65**	0.30	**0.81**	**0.91**	**0.91**	**0.80**
*IL- 4*	**0.37**	**0.65**	-	0.16	**0.65**	**0.73**	**0.68**	**0.58**
*IL-6*	-0.10	0.30	0.16	-	0.05	0.20	0.19	0.22
*IL-10*	**0.46**	**0.82**	**0.65**	0.05	-	**0.88**	**0.90**	**0.72**
*IFN-γ*	**0.54**	**0.92**	**0.74**	0.20	**0.88**	-	**0.95**	**0.80**
*TNF- α*	**0.50**	**0.92**	**0.68**	0.19	**0.90**	**0.95**	-	**0.80**
*TGF-β1*	**0.38**	**0.81**	**0.58**	0.22	**0.72**	**0.80**	**0.80**	-

### Multivariate analysis for the association between disease severity and different variables

Multivariable logistic regression performed using all variables from the data set with ‘outcome variable’, namely severity, in the mild/severe groups, showed a statistically significant association between age, *IL-6* expression, and severity (Table 3).

**Table 3:** A multivariable logistic regression model including all variables. The table shows the odds ratio (OR), information coefficient (IC), and 95% confidence interval (CI). Multivariable logistic regression: forward model, for only significant variables in the full analysis, is shown in the bottom half.

**Variables**	**OR**	**IC**	**95% CI**
***IL-1***	0.925	0.824	1.037
***IL-2***	1.227	0.900	1.674
***IL-4***	0.941	0.813	1.088
***IL-6***	1.083	1.039	1.128
***IL-10***	1.017	0.886	1.168
***IFN-γ***	1.208	0.873	1.671
***TNF- α***	1.053	0.811	1.367
***TGF-β1***	0.834	0.654	1.064
**AGE**	1.042	1.008	1.077
**GENDER**	1.080	0.345	3.381
**Viral load (Ct value)**	1.031	0.935	1.136
**Multivariable logistic regression: forward model**			
**Variables**	**OR**	**IC**	**95% CI**
***IL-6***	1.083	1.039	1.128
**AGE**	1.042	1.008	1.077

## Discussion

In this study, nasopharyngeal swab samples were obtained from patients with mild and severe COVID-associated symptoms and were used to study the viral loads and the differential expression of various cytokines in the samples and their association with the severity of the COVID-19 disease [19]. For the better management of the patients, the diagnosis and treatment guidelines for SARS-CoV-2-associated pneumonia recommend the monitoring of the cytokine expression to assess the progression of the disease [20]. It also highlights the need to identify the potential early biomarkers to identify those patients who might be on their way toward a severe form of the disease [21]. Therefore, we analyzed the viral loads and the differential gene expression profiles of eight cytokines (*IL-1*, *IL-2*, *IL-4*, *IL-6*, *IL-10 IFN-γ*, *TNF-α*, *and TGF-β1*) in a total of 118 qPCR confirmed COVID-19 cases. It is important to mention that this study is part of the larger study assessing immunological response against SARS-CoV-2 in HIV-positive and HIV-negative individuals. All samples used in this study were from HIV-negative individuals.

Although the high viral load has been associated with increased severity of the disease [22], however, we did not find a statistically significant difference in the viral loads of mild and severe groups. This finding is supported by other studies which also did not find a significant association between the clinical outcome of COVID-19 and viral load [23], and have shown that it is an uncontrolled immune response involving hypercytokinemia, and not just the high viral titers, which is associated with the adverse clinical outcomes in COVID-19 patients [24].

The differential expression of cytokines in the nasopharyngeal mucosa forms an important component of mucosal immunity against COVID-19 disease and many pro-inflammatory cytokines are secreted by epithelial cells to mediate immune response against the virus, where the optimal expression and activity of the anti-viral cytokines ensures viral clearance [25, 26]. Interestingly, in this study, the analysis of the differential expression of cytokines in the mild and severe COVID-19 patient groups revealed the expression of *IL-2*, *IL-6*, *TNF-α*, and *IFN-γ* to be significantly decreased in severe cases as compared to mild cases. In contrast, studies performed on serum samples had earlier reported higher IL-2, TNF-α, IFN-α, and IFN-γ levels in serum of patients with severe COVID-19 disease as compared to the mild or mild-moderate patients [19, 27–32]. *Boan Li et al*, while investigating the differential serum cytokine expression in individuals with severe and mild COVID-19 disease, reported that IL-6 was significantly increased in COVID-19 of the severe group as compared with the mild disease group [33]. This difference, observed in the current study, may be due to the type of patient samples used (serum vs nasal swabs) and suggest that the downregulation of the key cytokines, *IL-2*, *IL-6*, *TNF-α*, and *IFN-γ*, occurs in the nasopharyngeal milieu during the early infection. However, other studies have shown that some of the COVID-19 patients not only had higher levels of serum IL-6 but also the severe form of the disease was predicted by the imbalanced cytokine production [34]. Similarly, *Abers et al* [35] showed that elevated levels of IL-6 were independently related to high mortality in a study that examined the levels of 66 soluble biomarkers in patients with varying levels of COVID-19 severity. In addition, a recent meta-analysis suggested that the levels of IL-6 are associated with the severe form of the disease [36]. It is possible that the suppression of these cytokines (some being potent antiviral cytokines) in the nasopharyngeal mucosa may allow the propagation and the systemic dissemination of virus which may lead to systemic cytokine storm, resulting in the severe form of the disease.

This observation was further confirmed by our multivariate logistic regression model results showed that the severity of the disease was significantly associated with age and nasopharyngeal IL-6 expression. Age was found to be an independent factor in our study showing a significant association with the disease severity. Earlier studies on COVID-19 frequently associated disease severity with age, where elderly patients were found to experience a severe form of the disease [37].

In the correlation analysis in the severe group, we found a positive correlation between *IL-1* and *TNF-α*, *IFN- γ*, *TGF-β1; IL-2* and *IFN- γ*, *TNF-α*, *TGF-β1*; and *IL-4 and IFN- γ*, *TNF-α*, *TGF-β1; IL-10* and *IFN- γ*, *TNF-α*, *TGF-β1*, respectively. A previous study on acute-phase cytokines in the nasopharynx also reported cytokines *IL-1*, *IL-6*, and *TNF-α* to be positively correlated with each other [38]. Furthermore, studies on serum of COVID-19 patients have recommended assessing *TGF-α*, *IFN-γ*, *TGF-β*, *IL-1*, and *IL-10* because of their association with the severity of the disease [39–41], however, importantly the role of these immune mediators as part of nasopharyngeal mucosal immunity against COVID-19 has yet to be established.

We identify several limitations of our study. Firstly, in this study, we did not assess and adjust the role of other co-morbid conditions in mild and severe patients, which tend to have an important role in the clinical outcome of the disease [42]. Secondly, in our dataset, all age groups were represented, which may act as a confounder (age-related) for gene expression profiles of the cytokines in the nasopharyngeal mucosa [43]. Lastly, we only examined the gene expression profiles of the cytokine in nasopharyngeal mucosa, and levels of these cytokines in serum were not accessed due to the unavailability of serum samples, and, therefore, a relationship between cytokine expression in nasopharyngeal milieu and serum and disease severity cannot be established. However, this does not undermine our original aim to examine early cytokine changes in the nasopharyngeal mucosa and assess its possible role in predicting the severity of the disease.

In conclusion, we found the decreased nasopharyngeal expression of pro-inflammatory cytokines *IL-2*, *Il-6*, *IFN-γ*, and *TNF-α* to be significantly higher in the severe group as compared to the mild group. Early detection of the cytokine profile may be useful in predicting the development of the severe form of the disease in patients with COVID-19, and such patients can be provided with appropriate anti-viral therapy or monitored closely for development of severe symptoms.

## Supporting information

S1 Table List of primers for cytokine and β-actin genes used in the qPCR array. (DOCX)

S1 Data (XLSX)

## References

[1] Zhao D, Yao F, Wang L, Zheng L, Gao Y, Ye J, et al. A comparative study on the clinical features of COVID-19 pneumonia to other pneumonias. Clinical Infectious Diseases. 2020. 10.1093/cid/ciaa247 PMC7108162 32161968

[2] Huang C, Wang Y, Li X, Ren L, Zhao J, Hu Y, et al. Clinical features of patients infected with 2019 novel coronavirus in Wuhan, China. The lancet. 2020;395(10223):497–506. 10.1016/S0140-6736(20)30183-5 PMC7159299 31986264

[3] Park SH. An impaired inflammatory and innate immune response in COVID-19. Molecules and Cells. 2021;44(6):384. doi: 10.14348/molcells.2021.0068 34098591 PMC8245320

[4] Bajaj V, Gadi N, Spihlman AP, Wu SC, Choi CH, Moulton VR. Aging, immunity, and COVID-19: how age influences the host immune response to coronavirus infections? Frontiers in Physiology. 2021;11:1793. doi: 10.3389/fphys.2020.571416 33510644 PMC7835928

[5] Cai C, Tang Y-D, Xu G, Zheng C. The crosstalk between viral RNA-and DNA-sensing mechanisms. Cellular and Molecular Life Sciences. 2021;78(23):7427–34. doi: 10.1007/s00018-021-04001-7 34714359 PMC8554519

[6] De Wit E, Van Doremalen N, Falzarano D, Munster VJ. SARS and MERS: recent insights into emerging coronaviruses. Nature Reviews Microbiology. 2016;14(8):523–34. doi: 10.1038/nrmicro.2016.81 27344959 PMC7097822

[7] Chilakamarti R. Profiling transcription factor sub-networks in type I interferon signaling and in response to SARS-CoV-2 infection. bioRxiv. 2021.

[8] Kishimoto K, Wilder CL, Buchanan J, Nguyen M, Okeke C, Hoffmann A, et al. High Dose IFN-β Activates GAF to Enhance Expression of ISGF3 Target Genes in MLE12 Epithelial Cells. Frontiers in immunology. 2021;12:1107. 10.3389/fimmu.2021.651254 PMC8062733 33897699

[9] Beacon TH, Su RC, Lakowski TM, Delcuve GP, Davie JR. SARS‐CoV‐2 multifaceted interaction with the human host. Part II: Innate immunity response, immunopathology, and epigenetics. IUBMB life. 2020;72(11):2331–54. doi: 10.1002/iub.2379 32936531

[10] Peiris JSM, Chu C-M, Cheng VC-C, Chan K, Hung I, Poon LL, et al. Clinical progression and viral load in a community outbreak of coronavirus-associated SARS pneumonia: a prospective study. The Lancet. 2003;361(9371):1767–72. 10.1016/S0140-6736(03)13412-5 PMC7112410 12781535

[11] Wong C, Lam C, Wu A, Ip W, Lee N, Chan I, et al. Plasma inflammatory cytokines and chemokines in severe acute respiratory syndrome. Clinical & Experimental Immunology. 2004;136(1):95–103. doi: 10.1111/j.1365-2249.2004.02415.x 15030519 PMC1808997

[12] Sierra B, Pérez AB, Aguirre E, Bracho C, Valdés O, Jimenez N, et al., editors. Association of early nasopharyngeal immune markers with COVID-19 clinical outcome: predictive value of CCL2/MCP-1. Open forum infectious diseases; 2020: Oxford University Press US. doi: 10.1093/ofid/ofaa407 33123608 PMC7499702

[13] Chi Y, Ge Y, Wu B, Zhang W, Wu T, Wen T, et al. Serum cytokine and chemokine profile in relation to the severity of coronavirus disease 2019 in China. The Journal of infectious diseases. 2020;222(5):746–54. doi: 10.1093/infdis/jiaa363 32563194 PMC7337752

[14] Son KB, Lee TJ, Hwang SS. Disease severity classification and COVID-19 outcomes, Republic of Korea. Bull World Health Organ. 2021;99(1):62–6. Epub 2021/03/05. doi: 10.2471/BLT.20.257758 ; PubMed Central PMCID: PMC7924894. 33658735 PMC7924894

[15] WHO. COVID-19 therapeutic trial synopsis. Geneva: World Health Organization Feb, 18,2020 [cited https://www.who.int/blueprint/prioritydiseases/key-action/COVID-19_Treatment_Trial_Design_Master_Protocol_synopsis_Final_18022020.pdf (accessed April 25, 2020)].

[16] Ahmed MA, Anwar MF, Ahmed K, Aftab M, Nazim F, Bari MF, et al. Baseline MMP expression in periapical granuloma and its relationship with periapical wound healing after surgical endodontic treatment. BMC Oral Health. 2021;21(1):1–9. 34732191 10.1186/s12903-021-01904-6 PMC8565031

[17] Schmittgen TD, Livak KJ. Analyzing real-time PCR data by the comparative C(T) method. Nat Protoc. 2008;3(6):1101–8. Epub 2008/06/13. doi: 10.1038/nprot.2008.73 . 18546601

[18] Livak KJ, Schmittgen TD. Analysis of relative gene expression data using real-time quantitative PCR and the 2(-Delta Delta C(T)) Method. Methods. 2001;25(4):402–8. Epub 2002/02/16. doi: 10.1006/meth.2001.1262 . 11846609

[19] Monel B, Planas D, Grzelak L, Smith N, Robillard N, Staropoli I, et al. Release of infectious virus and cytokines in nasopharyngeal swabs from individuals infected with non-alpha or alpha SARS-CoV-2 variants: an observational retrospective study. EBioMedicine. 2021;73:103637. doi: 10.1016/j.ebiom.2021.103637 34678613 PMC8526063

[20] Sun X, Wang T, Cai D, Hu Z, Liao H, Zhi L, et al. Cytokine storm intervention in the early stages of COVID-19 pneumonia. Cytokine & growth factor reviews. 2020;53:38–42. doi: 10.1016/j.cytogfr.2020.04.002 32360420 PMC7182527

[21] Nagant C, Ponthieux F, Smet J, Dauby N, Doyen V, Besse-Hammer T, et al. A score combining early detection of cytokines accurately predicts COVID-19 severity and intensive care unit transfer. International Journal of Infectious Diseases. 2020;101:342–5. doi: 10.1016/j.ijid.2020.10.003 33039609 PMC7544772

[22] Fajnzylber J, Regan J, Coxen K, Corry H, Wong C, Rosenthal A, et al. SARS-CoV-2 viral load is associated with increased disease severity and mortality. Nature communications. 2020;11(1):1–9. 10.1038/s41467-020-19057-5 PMC7603483 33127906

[23] Abdulrahman A, Mallah SI, Alqahtani M. COVID-19 viral load not associated with disease severity: findings from a retrospective cohort study. BMC infectious diseases. 2021;21(1):1–4. 34271860 10.1186/s12879-021-06376-1 PMC8284033

[24] Argyropoulos KV, Serrano A, Hu J, Black M, Feng X, Shen G, et al. Association of initial viral load in severe acute respiratory syndrome coronavirus 2 (SARS-CoV-2) patients with outcome and symptoms. The American journal of pathology. 2020;190(9):1881–7. doi: 10.1016/j.ajpath.2020.07.001 32628931 PMC7332909

[25] Moradi-Kalbolandi S, Majidzadeh-A K, Abdolvahab MH, Jalili N, Farahmand L. The role of mucosal immunity and recombinant probiotics in SARS-CoV2 vaccine development. Probiotics and antimicrobial proteins. 2021;13(5):1239–53. doi: 10.1007/s12602-021-09773-9 33770348 PMC7996120

[26] Pierce CA, Sy S, Galen B, Goldstein DY, Orner E, Keller MJ, et al. Natural mucosal barriers and COVID-19 in children. JCI insight. 2021;6(9). 10.1172/jci.insight.148694 PMC8262299 33822777

[27] Chen L-D, Zhang Z-Y, Wei X-J, Cai Y-Q, Yao W-Z, Wang M-H, et al. Association between cytokine profiles and lung injury in COVID-19 pneumonia. Respiratory Research. 2020;21(1):1–8. 32727465 10.1186/s12931-020-01465-2 PMC7389162

[28] Rouchka EC, Chariker JH, Alejandro B, Adcock RS, Singhal R, Ramirez J, et al. Induction of interferon response by high viral loads at early stage infection may protect against severe outcomes in COVID-19 patients. Scientific reports. 2021;11(1):1–14. 34344959 10.1038/s41598-021-95197-y PMC8333042

[29] Mondi A, Cimini E, Colavita F, Cicalini S, Pinnetti C, Matusali G, et al. COVID‐19 in people living with HIV: Clinical implications of dynamics of the immune response to SARS‐CoV‐2. Journal of Medical Virology. 2021;93(3):1796–804. doi: 10.1002/jmv.26556 32975842 PMC7537181

[30] Mohanty MC, Varose SY, Sawant UP, Fernandes MM. Expression of innate immune response genes in upper airway samples of SARS-CoV-2 infected patients: A preliminary study. The Indian journal of medical research. 2021;153(5–6):677. doi: 10.4103/ijmr.IJMR_131_21 34528526 PMC8555587

[31] Cameron MJ, Bermejo-Martin JF, Danesh A, Muller MP, Kelvin DJ. Human immunopathogenesis of severe acute respiratory syndrome (SARS). Virus research. 2008;133(1):13–9. doi: 10.1016/j.virusres.2007.02.014 17374415 PMC7114310

[32] Theron M, Huang K-J, Chen Y-W, Liu C-C, Lei H-Y. A probable role for IFN-γ in the development of a lung immunopathology in SARS. Cytokine. 2005;32(1):30–8. 16129616 10.1016/j.cyto.2005.07.007 PMC7129778

[33] Li B, Feng F, Yang G, Liu A, Yang N, Jiang Q, et al. Immunoglobulin G/M and cytokines detections in continuous sera from patients with novel coronaviruses (2019-nCoV) infection. Available at SSRN 3543609. 2020.

[34] Hashizume M. Outlook of IL-6 signaling blockade for COVID-19 pneumonia. Inflammation and Regeneration. 2020;40(1):1–8. doi: 10.1186/s41232-020-00134-7 33024459 PMC7533147

[35] Abers MS, Delmonte OM, Ricotta EE, Fintzi J, Fink DL, de Jesus AAA, et al. An immune-based biomarker signature is associated with mortality in COVID-19 patients. JCI insight. 2021;6(1). doi: 10.1172/jci.insight.144455 33232303 PMC7821609

[36] Akbari H, Tabrizi R, Lankarani KB, Aria H, Vakili S, Asadian F, et al. The role of cytokine profile and lymphocyte subsets in the severity of coronavirus disease 2019 (COVID-19): a systematic review and meta-analysis. Life sciences. 2020;258:118167. doi: 10.1016/j.lfs.2020.118167 32735885 PMC7387997

[37] Ebinger JE, Achamallah N, Ji H, Claggett BL, Sun N, Botting P, et al. Pre-existing traits associated with Covid-19 illness severity. PloS one. 2020;15(7):e0236240. doi: 10.1371/journal.pone.0236240 32702044 PMC7377468

[38] Patel JA, Nair S, Revai K, Grady J, Chonmaitree T. Nasopharyngeal acute phase cytokines in viral upper respiratory infection: impact on acute otitis media in children. The Pediatric infectious disease journal. 2009;28(11):1002. doi: 10.1097/INF.0b013e3181aa5b13 19859015 PMC3220942

[39] Lu Q, Zhu Z, Tan C, Zhou H, Hu Y, Shen G, et al. Changes of serum IL‐10, IL‐1β, IL‐6, MCP‐1, TNF‐α, IP‐10 and IL‐4 in COVID‐19 patients. International Journal of Clinical Practice. 2021;75(9):e14462. 34107113 10.1111/ijcp.14462 PMC8237069

[40] Colarusso C, Maglio A, Terlizzi M, Vitale C, Molino A, Pinto A, et al. Post-COVID-19 patients who develop lung fibrotic-like changes have lower circulating levels of IFN-β but higher levels of IL-1α and TGF-β. Biomedicines. 2021;9(12):1931. 34944747 10.3390/biomedicines9121931 PMC8698335

[41] Ghazavi A, Ganji A, Keshavarzian N, Rabiemajd S, Mosayebi G. Cytokine profile and disease severity in patients with COVID-19. Cytokine. 2021;137:155323. doi: 10.1016/j.cyto.2020.155323 33045526 PMC7524708

[42] Sanyaolu A, Okorie C, Marinkovic A, Patidar R, Younis K, Desai P, et al. Comorbidity and its impact on patients with COVID-19. SN comprehensive clinical medicine. 2020;2(8):1069–76. doi: 10.1007/s42399-020-00363-4 32838147 PMC7314621

[43] Fröberg J, Diavatopoulos DA. Mucosal immunity to severe acute respiratory syndrome coronavirus 2 infection. Current Opinion in Infectious Diseases. 2021;34(3):181–6. doi: 10.1097/QCO.0000000000000724 33899752

