# Supplementary material for: Analysis of differential gene expression of pro-inflammatory cytokines in the nasopharyngeal milieu of mild & severe COVID-19 cases
Source: PLoS One. 2022 Dec 30;17(12):e0279270. doi: 10.1371/journal.pone.0279270 (PMC9803207; doi:10.1371/journal.pone.0279270)
Supplement: S1 Table — (DOCX) [file pone.0279270.s001.docx]

**S1 Table:** List of primers for cytokine and β-actin genes used in the qPCR array.

| **Genes** | **Forward Primer (5’—3’)** | **Reverse Primer (3’—5’)** |
| --- | --- | --- |
| ***IL- 1*** | 5-ATGATGGCTTACSGTGGCAA-3 | 3-GTCGGAGATTCGTAGCTGGA-5 |
| ***IL-2*** | 5-GAAGATCGTCATGGGAAGGAAGC-3 | 3-CGGGTATTTATAGTGGCATGGG-5 |
| ***IL- 4*** | 5- CCAACTGCTTCCCCCTCTG-3 | 3-TCTGTTACGGTCAACTCGGTG-5 |
| ***IL-6*** | 5- ACTCACCTCTTCAGAACGAATTG-3 | 3-CCATCTTTGGAAGGTTCAGGTTG-5 |
| ***Il-10*** | 5-GACTTTAAGGGTTACCTGGGTTG-5 | 3-TCACATGCGCCTTGATGTCTG-5 |
| ***IFN-γ*** | 5-GAGGCCAAGCCCTGGTATG-3 | 3- CGGGCCGATTGATCTCAGC-5’ |
| ***TNF-***$\boldsymbol{\propto}$ | 5- TCGGTAACTGACTTGAATGTCCA-3 | `3- TCGCTTCCCTGTTTTAGCTGC-`5 |
| ***TGF-β1*** | 5-CAATTCCTGGCGATACCTCAG-3 | 3-GCACAACTCCGGTGACATCAA-5 |
| ***B-actin*** | 5-CAACTTCATCCAGCTTCACC-3 | 3-TCGAGGACGCCCTATCATGG-5 |
